# Changes in Brain Neuroimmunology Following Injury and Disease

**DOI:** 10.3389/fnint.2022.894500

**Published:** 2022-04-27

**Authors:** Anthony Tabet, Caroline Apra, Alexis M. Stranahan, Polina Anikeeva

**Affiliations:** ^1^McGovern Institute for Brain Research, Massachusetts Institute of Technology, Cambridge, MA, United States; ^2^Research Laboratory of Electronics, Massachusetts Institute of Technology, Cambridge, MA, United States; ^3^Department of Chemical Engineering, Massachusetts Institute of Technology, Cambridge, MA, United States; ^4^Sorbonne Universite, Paris, France; ^5^Department of Neuroscience and Regenerative Medicine, Augusta University, Augusta, GA, United States; ^6^Department of Materials Science and Engineering, Massachusetts Institute of Technology, Cambridge, MA, United States; ^7^Department of Brain and Cognitive Sciences, Massachusetts Institute of Technology, Cambridge, MA, United States

**Keywords:** extracellular matrix, blood-brain barrier, neuro-immunology, contusions, stroke, glioblastoma, multiple sclerosis

## Abstract

The nervous and immune systems are intimately related in the brain and in the periphery, where changes to one affect the other and vice-versa. Immune cells are responsible for sculpting and pruning neuronal synapses, and play key roles in neuro-development and neurological disease pathology. The immune composition of the brain is tightly regulated from the periphery through the blood-brain barrier (BBB), whose maintenance is driven to a significant extent by extracellular matrix (ECM) components. After a brain insult, the BBB can become disrupted and the composition of the ECM can change. These changes, and the resulting immune infiltration, can have detrimental effects on neurophysiology and are the hallmarks of several diseases. In this review, we discuss some processes that may occur after insult, and potential consequences to brain neuroimmunology and disease progression. We then highlight future research directions and opportunities for further tool development to probe the neuro-immune interface.

## 1. Brain Extracellular Matrix and the Blood-Brain Barrier

The extracellular matrix (ECM) is an integral component contributing to brain function during health and disease (Baeten and Akassoglou, 2011). The ECM affects neuronal and glial function through delivery of nutrients, regulation of signaling molecules, and maintenance of overall stiffness (Baeten and Akassoglou, 2011; Jakeman et al., 2014; Segel et al., 2019; Tabet et al., 2019a). The ECM is also integral to the blood-brain barrier (BBB), which regulates the transport of molecules and cells between peripheral blood and the central nervous system (CNS).

**Figure 1:**
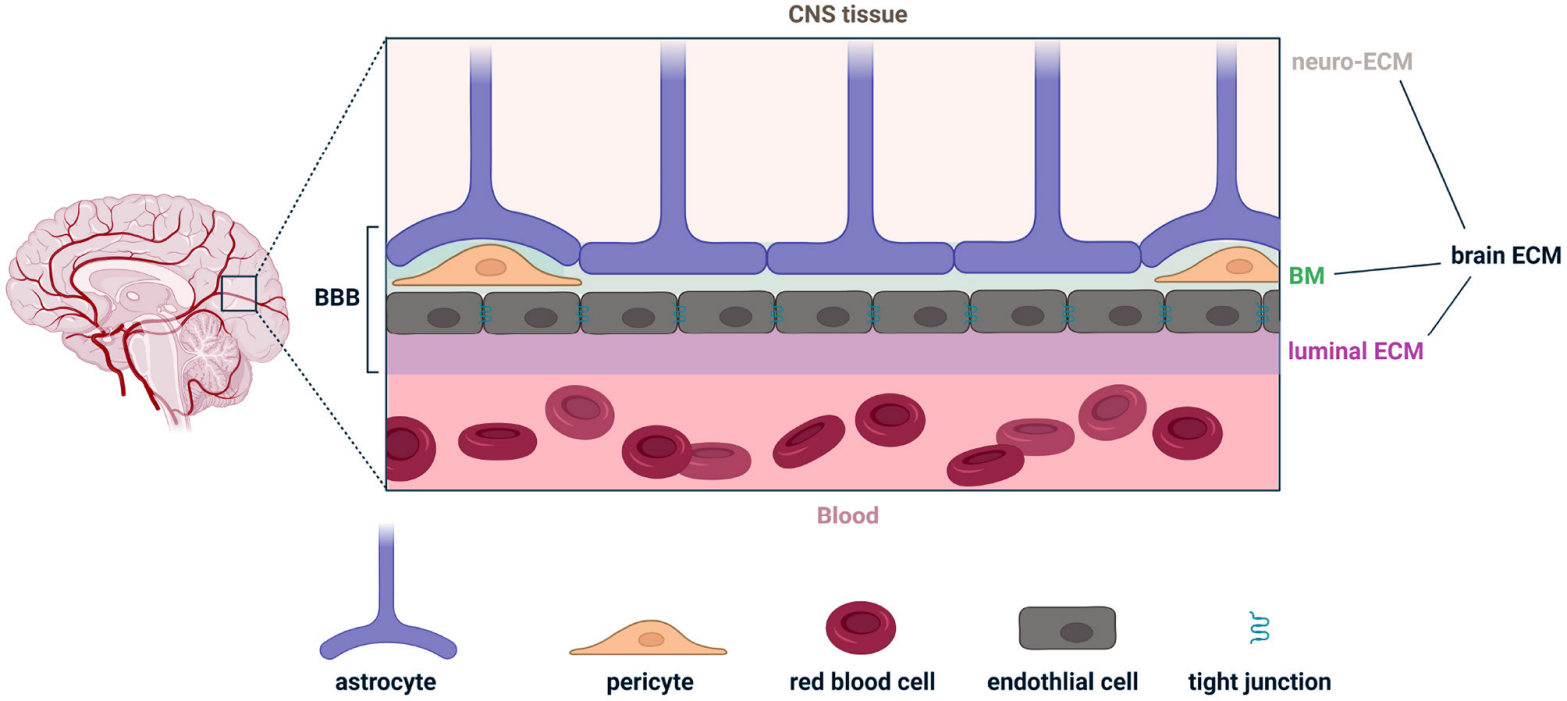
Illustration of the various components comprising the brain extracellular matrix (brain ECM) and the blood-brain barrier (BBB). The brain ECM is the combination of the ECM in the central nervous system (neuro-ECM), the basement membrane (BM), and the luminal ECM (also called the glycocalyx). The BBB is the combination of the BM, luminal ECM, and astrocytes, pericytes, and endothelial cells. Figure made with Biorender.

The extracellular matrix of the brain is composed of three parts: (1) the neuro-ECM, which is the conjunctive tissue of the brain and provides an active scaffold for neurons and glia; (2) the basement membrane (BM), which also constitutes the inner part of the BBB; and (3) the luminal ECM (synonymously called the glycocalyx), which interfaces with peripheral blood and is also the intravascular component of the BBB (Figure 1). The BBB includes extracellular components in the BM and luminal ECM, as well as cellular components such as endothelial cells, pericytes and astrocytes (Figure 1). In both the white and gray matter, the neuro-ECM is heavily populated by glycans such as hyaluronic acid (HA) and chondroitin sulfate (CS) (Tabet et al., 2019a,b). The neuro-ECM undergoes dramatic changes during development but remains relatively stable throughout life in the absence of disease. To support neuronal electrical activity, the neuro-ECM is organized in dense ECM structures known as perineuronal nets which are enriched in HA/CS glycans, tenacin-R, and different combinations of lecticans, all secured by the link proteins Crtl1 and BraI2 (Bekku et al., 2010). Perineuronal nets enhance synaptic efficacy, restrict aberrant neuronal and synaptic reorganization, and protect neurons from metabolic stress (Wang and Fawcett, 2012; Cabungcal et al., 2013; Jakeman et al., 2014; Sorg et al., 2016).

The BM, a 40–100 nm thick ECM layer, anatomically separates endothelial cells from astrocyte endfeet (also known as glia limitans). It includes pericytes and is a composite of major ECM proteins: laminins, collagen IV, fibronectin, nidogens, and heparan sulfate proteoglycans. Laminins polymerize and provide a net for nidogens and heparan sulfate proteoglycans to bind to. Collagen IV provides a stable scaffold for the matrix (Sood et al., 2016; Linka et al., 2021). Last, fibronectin, which incorporates into the matrix and facilitates its organization, directly affects the barrier properties and stimulates the proliferation and survival of endothelial cells (Reed et al., 2019).

The luminal ECM is thicker than the BM by an order of magnitude, ranging from 0.2 to 5 μm. Unlike the neuro-ECM and the BM, the luminal ECM is in permanent contact with blood, is subject to more environmental stressors, and is directly exposed to peripheral immune cells circulating in plasma. HA, which can be produced by endothelial cells and astrocytes, is a key driver of luminal ECM permeability across the BBB (Henry and Duling, 1999). HA interacts with cells *via* receptors such as CD44 and can trigger an inflammatory cascade (Misra et al., 2015). HA can also mitigate inflammation *via* interaction with hyaladherins (Lesley et al., 2004; Reed et al., 2019). The molecular weight (MW) of HA can dramatically alter its immunological properties, where low MW is pro-inflammatory and high MW is anti-inflammatory (Rayahin et al., 2015). Interestingly, eliminating HA from the BBB increases its permeability for large molecules (Kutuzov et al., 2018). These various components of the extracellular space contribute to and regulate brain immunosurveillance in health, and are implicated in various disease pathologies that result in the breakdown of the BBB.

## 2. Immunosurveillance and Brain Immune Homeostasis

Classical concepts surrounding separation of the brain and the peripheral immune system are gradually giving way to a more dynamically regulated interface, with ECM components gating inter-cellular interactions and where astrocytes, pericytes, and endothelial cells play an outsized role in BBB maintenance (Figure 1). Microglia, the resident macrophages of the brain, play a key role in immunosurveillance and are also important to BBB integrity. Similar to resident macrophages in the periphery, microglia are sentinel cells that survey the brain during homeostasis. They can also be polarized to respond to CNS insults and support wound healing. Microglia have received increasing attention for their role in regulation of BBB integrity and the underlying mechanisms through which they amplify or suppress circulating factors transported into the brain parenchyma (Lou et al., 2016; Guo et al., 2020). Extracellular purines are potent attractants for microglia and for other tissue-resident myeloid cells (Idzko et al., 2014; Badimon et al., 2020). Recent *in vivo* imaging experiments revealed that after damage to blood vessels in the brain, microglia traffic to the site of injury and prevent further vascular leakage by forming a dense aggregate of microglial processes (Lou et al., 2016). This is one elegant example of dynamic interactions between microglia and the BBB which illustrates their role in immunosurveillance and homeostasis (Zhao et al., 2015; Thurgur and Pinteaux, 2019).

A rapidly growing field of research is exploring brain immunosurveillance from non-microglial cells, including other myeloid cells and lymphocytes. The meninges have emerged as a critical reservoir of innate and adaptive immune cells, and meningeal lymphatics play critical roles in brain antigen drainage (Louveau et al., 2015, 2018; Lima et al., 2020; Papadopoulos et al., 2020; Brioschi et al., 2021). Bone marrow niches in the skull were shown to provide meninges with a continuous supply of myeloid cells through bone marrow–dura channels (Cugurra et al., 2021). Following this study, it was additionally shown proteins in the CSF provide cues for myelopoiesis and cell-trafficking to the meninges (Mazzitelli et al., 2022). Lymphatics in the meninges are also critical for brain immunosurveillance by T cells (Louveau et al., 2015, 2018). Improving lymphatic drainage with vascular endothelial growth factor C (VEGF-C) (Mesquita et al., 2018), for example, can enhance T cell-based immunotherapy against gliomas (Song et al., 2020). VEGF-C can also improve monoclonal antibody treatments against amyloid beta in murine models of Alzheimer's disease (Mesquita et al., 2021). Another exciting area of research explores the effects of innate or adaptive immune cells on neuronal function. The former secrete soluble cytokines into the ECM, which can directly bind to receptors on neurons (Salvador et al., 2021).

Still, many questions remain, and the field faces substantial technological challenges. Identifying the points of entry for perturbation of inter-cellular communication in disease states will require novel strategies for spatiotemporal control of individual cells and the surrounding matrix. Studies in the cortex have been more accessible than in deeper structures. For example, intravital imaging approaches have been adapted for studies of leukocyte rolling and adhesion in penetrating arteries on the surface of the cortex. While downstream capillaries are currently less accessible for kinetic imaging, the development of implantable bioelectronics for stealthy interrogation of intracerebral processes (Canales et al., 2015; Liang et al., 2018; Tabet et al., 2021) is a promising strategy to access deeper structures. Imaging deeper into the brain could reveal new modes of interaction between circulating lymphocyte precursors and cell populations at the BBB and blood-cerebrospinal fluid interfaces, with downstream consequences for healthy neuronal processes and disease interventions.

## 3. Changes Following CNS Insult

**Figure 2:**
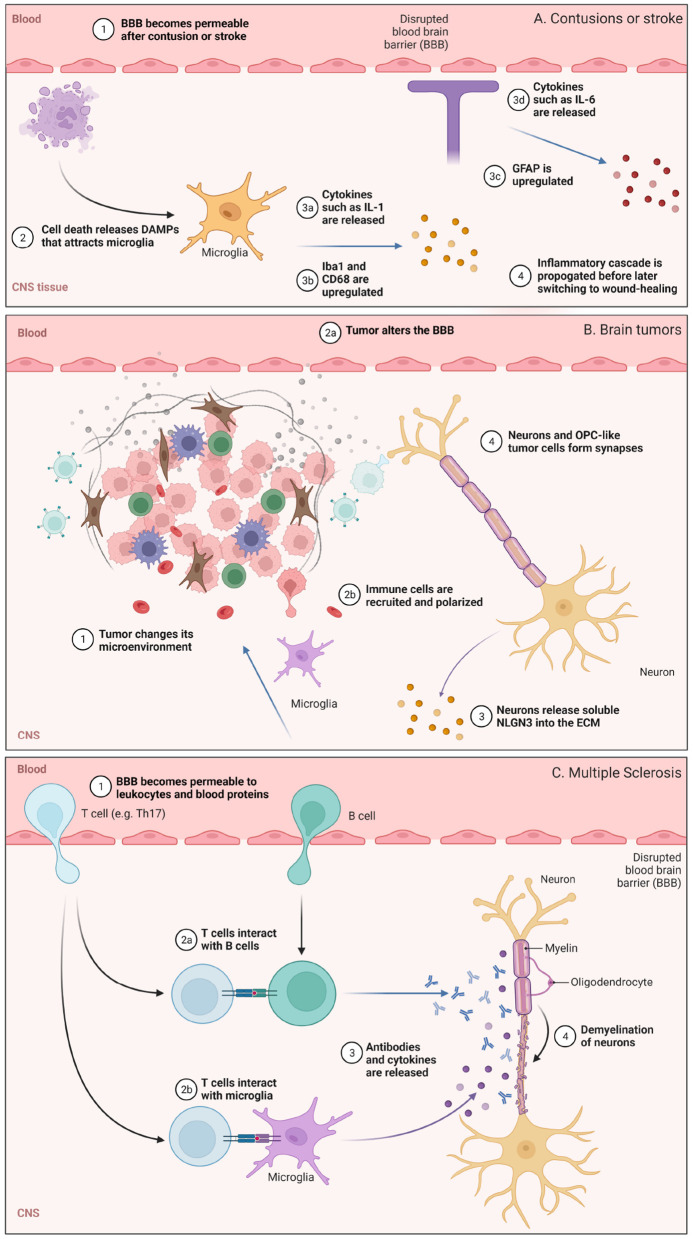
Illustration highlighting some of the cellular or molecular pathways involved in **(A)** contusions or stroke, **(B)** glioblastoma (GB), and **(C)** multiple sclerosis (MS), with events that occur under comparable time scales grouped together. **(A)** BBB permeabilization and the role of microglia are highlighted. **(B)** Changes in the brain tumor microenvironment and some neuro-immune consequences. **(C)** Lymphocyte trafficking into the CNS and subsequent demyelination in MS. Figure made with Biorender.

Dramatic changes to the BBB, ECM, and brain immunosurveillance can occur after an insult to the CNS. These insults, such as cortical contusion, stroke, glioblastoma (GB), and multiple sclerosis (MS) occur over varying timescales and can have different consequences on microglia, astrocytes, and lymphocytes (Figure 2). We discuss some features of these pathologies below.

### 3.1. Contusions and Stroke

Cortical contusions are traumatic lesions in the brain that hemorrhage. Following injury, cellular and macromolecular components in blood can traverse the disrupted BBB and enter the CNS. This damage triggers an array of signaling cascades that activate the innate immune system (Jakeman et al., 2014). Dying cells release genomic DNA and other damage-associated molecular patterns (DAMPs; Vénéreau et al., 2015) which serve as biochemical cues (Figure 2A). Microglia sense these DAMPs and are polarized toward phagocytic, pro-inflammatory phenotypes which upregulate membrane-bound CD68 and release signaling molecules such as IL-1, IL-6, and TNF-α. These activated microglia also upregulate ionized calcium binding adaptor molecule 1 (Iba1; Ohsawa et al. (2004)), a protein that participates in microglia phagocytosis. As a response to brain injury, astrocytes also undergo major changes and will upregulate glial fibrillary acidic protein (GFAP) as well as release cytokines and chemokines to attract microglia, circulating monocytes, and other immune cells from the periphery to the site of injury. Interestingly, inflammatory peptides such as bradykinin can cause astrocytes to secrete IL-6 and lead to further BBB permeabilization early on, suggesting that temporarily heightened peripheral immune surveillance supports an inflammatory response before astrocyte-mediated repair of the BBB (Schwaninger et al., 1999; Karve et al., 2016). Following cytokine and chemokine release soon after injury, peripheral immune cells such as neutrophils and circulating monocytes enter the brain. These circulating monocytes differentiate into macrophages and can persist for several days to weeks (Jakeman et al., 2014; Jin and Yamashita, 2016) while exhibiting both pro-inflammatory and wound-healing phenotypes.

Dramatic changes to the ECM occur following contusions. Following stimulation by signaling molecules such as IL-6, IFNγ, and TGFβ, astrocytes secrete chondroitin sulfate (CS) and CS-containing proteoglycans (CSPGs). CS levels increase 1–7 days following injury, and can remain elevated for weeks before reduction ~2 months later (Jakeman et al., 2014); they also have a profound effect on neurons (Siebert et al., 2014). CSPGs inhibit axonal growth, and upregulation of CS *in vivo* by astrocytes in the area of injury results in a reduction of regenerative capacity. This self-defense mechanism may be an endogenous way to wall off injury and prevent its propagation, and controlling it is an area of research for regenerative medicine (Jakeman et al., 2014; Siebert et al., 2014).

Strokes are the second leading cause of death worldwide (WHO Global Health Estimates), and preventing them or limiting their damage has been the focus of extensive research. Strokes are disruptions of blood flow to the brain, triggered either by a blood clot causing ischemia in about 80% of cases (Aguilar, 2015), or by hemorrhagic vessel disruptions. This paragraph will focus on the consequences of ischemic strokes, which share some immune cascades with contusions.

Ischemia leads to the disruption of the BBB through several mechanisms (Baeten and Akassoglou, 2011). At the cellular level, ischemia causes a disruption of tight junctions between endothelial cells, with subsequent opening of the endothelial and astrocytic end feet barriers. Ischemia affects the ECM as well by causing a degradation of the BM due to proteolysis by matrix metalloproteases (MMPs). Fibrin and fibrinogen extravasation from the bloodstream through the disrupted BBB leads to secondary damage in the brain as well.

As with contusions, this damage in the brain from ischemia triggers an orchestrated neuro-inflammatory cascade that begins with an influx of microglia to the site of injury. Soluble factors released as a consequence of the cell injury in the parenchyma or from blood extravasation activate microglia (Qin et al., 2019). These activated microglia can play multiple roles at the BBB in ischemic stroke. Starting in the initial hours after ischemic stroke and peaking a week after, microglia produce high levels of pro-inflammatory cytokines and chemokines, which enhance the infiltration of T and B leukocytes (Selvaraj and Stowe, 2017; Zhang et al., 2021). In latter stages, microglia exhibit wound-healing phenotypes and support synpatic remodeling. They release VEGF, an angiogenic factor, to promote reformation of vasculature. Microglia also triggers expression of synaptic proteins and plays a role in neurogenesis, axonal regeneration, and other neural circuit recovery processes (Yu et al., 2021).

Following the influx of microglia, other cellular players traffic to the site of injury. In addition to circulating macrophages, T and B lymphocytes, dendritic cells (DCs), and neutrophils enter the brain and play a role in the neuro-inflammatory and healing cascades that follow (Selvaraj and Stowe, 2017). The role of the adaptive immune system in recovery following ischemia is still not fully understood. Interestingly, in animal models of stroke, wild-type mice fare worse than immune-deficient mice, and IFN-γ and IL-17 have been identified as key players in T cell-mediated tissue damage (Yilmaz et al., 2006; Shichita et al., 2009; Gelderblom et al., 2012; Selvaraj and Stowe, 2017 The long-term effects of adaptive immune cell influx into the brain following stroke remain an active area of research. Further investigations into their acute and chronic functions, and interactions with neurons, could open up new therapeutic strategies that address this large unmet medical need.

### 3.2. Glioblastoma

Glioblastomas (GBs) are aggressive grade IV astrocytomas and are the most common primary intracranial tumors (Bondy et al., 2008). GBs are universally lethal and have among the worst clinical outcomes of all cancer types. They develop a tumor microenvironment with altered cellular and extracellular composition that is crucial to their progression and therapeutic resistance, characterized by metabolic, hypoxic, osmotic and pH changes (Qiu et al., 2021). The neuro-ECM in this tumor environment supports cancer stem cells and is involved in tumor progression and chemoresistance (Ros et al., 2018) (Figure 2B). It provides receptors able to anchor stem cells in the niche and secretes growth factors that lead to their proliferation, such as laminin alpha-2 (Lathia et al., 2012), or regulation, such as integrin alpha-6 (Corsini and Martin-Villalba, 2010).

GBs cause BBB disruption, resulting in cerebral edema and tumor microenvironment communication with the blood stream (Wiranowska et al., 1992; Schneider et al., 2004). Disruption is not merely due to astrocytic dysfunction, but also due to GB cells secreting ECM-disrupting soluble factors (Schneider et al., 2004), for instance hypoxia-induced VEGF-A, also responsible for angiogenic stimulation (Zhao et al., 2018). They lead to a lower expression of claudin-1, claudin-5, and occludin, resulting in pathological fenestration in endothelial cells tight junctions, and increase the secretion of MMPs by endothelial cells, thus disrupting the ECM (Ishihara et al., 2008; Zhao et al., 2018).

However, the GB-induced BBB disruption, associated with the concept of a blood-brain tumor barrier, does not enable higher permeability of therapeutic macromolecules to the tumor. On the contrary, it creates a local microenvironment favorable for tumor growth and resistance against a variety of therapeutic modalities (Ros et al., 2018). The neuro-ECM is partly responsible for the increased stiffness of glioblastoma tissue and ECM compared to non-tumoral tissue, which, along the local hyperosmotic character, interferes with vessel integrity and is a significant obstacle to macromolecule transport across the BBB and the recruitment of inflammatory cells.

Brain tumors also have direct immuno-modulatory potential. Tumors are commonly thought of as “continuous wounds” with respect to their immunological characteristics and similarities of tumor-associated immune cells to those cells participating in wound-healing (Dvorak, 2015). GBs recruit immune cells by releasing soluble factors such as chemokines and cytokines, and can polarize them toward pro-tumor phenotypes. For example, microglia and peripheral monocytes/macrophages can be recruited and polarized to tumor-associated microglia (TAMs), and strikingly, these cells make up approximately 40% of a brain tumor's volume (Buonfiglioli and Hambardzumyan, 2021). TAMs are critical for tumor growth. They help reformulate the ECM to become more suitable for cancer cells, suppress effector immune cell surveillance, and enable brain tumor resistance to chemo- or radio-therapy (Buonfiglioli and Hambardzumyan, 2021).

The ability of neurons to contribute to tumor progression by secreting soluble factors and directly forming synapses with tumor cells has recently gained significant attention (Venkatesh et al., 2015, 2017, 2019; Venkataramani et al., 2019; Zeng et al., 2019; Monje et al., 2020; Pan et al., 2021). Of particular importance is a subset of glioma cells that resemble oligodendrocyte precursor cells (OPCs), referred to here as OPC-like cells (Neftel et al., 2019). When exposed to soluble forms of the neuronal synaptic protein neuroligin-3 (NLGN3), OPC-like tumor cells proliferated faster (Venkatesh et al., 2015) (Figure 2B). An experiment *in vitro* showed that tumor proliferation monotonically increased with increasing NLGN3 concentrations. Strinkingly, patient-derived cancer cells were not able to appreciably grow *in vivo* in Nlgn3-deficient mice. These data highlighted that soluble factors derived from neurons can modulate the growth of tumors, and preventing the cleavage of this post-synaptic protein is a viable therapeutic approach to reduce the rate of tumor progression (Venkatesh et al., 2017).

In follow-up studies, it was identified that OPC-like tumor cells form *bona fide* synapses with neurons *in vivo* (Venkataramani et al., 2019; Venkatesh et al., 2019). Serving as post-synaptic cells, OPC-like tumor cells had increased proliferation with higher activity independent of the soluble NLGN3 mechanism. *Ex vivo* slice patch-clamp experiments identified that synaptic communication occurs through AMPA receptors (AMPARs) and could be inhibited by traditional AMPAR antagonists such as NBQX or sodium channel blockers such as tetrodotoxin (TTX). A distinct, prolonged (>1 s) electrophysiological response was also observed *in vivo* that was not blocked by either AMPAR inhibitors or TTX. This was identified as a potassium current from a gap junction-coupled network, and this depolarization also promoted tumor growth. An unanswered question in this paradigm is whether TAMs have a role in neuron-tumor interactions. Given the role of microglia in sculpting neuronal synapses in healthy postnatal brains, an interesting question is whether TAMs support the formation of neuron-tumor synapses as well.

### 3.3. Multiple Sclerosis

Multiple sclerosis (MS) is a leading cause of neurological disability, particularly in young adults (WHO Global Health Estimates), and is caused by auto-immune destruction of the myelin sheath around neurons of the CNS. It impedes all types of cerebral and spinal chord functions, including motricity, sensitivity, vision and cognition. MS is currently incurable, though there are approved treatments that slow down disease progression (Martin et al., 2016).

Demyelination and oligodendrocyte loss are the primary pathological hallmarks in MS (Figure 2C). Peripheral leukocytes contribute to this pathology, where coordinated attacks involving misrecognition of myelin, antigen presentation, antibody production, and phagocytic or lytic attack of CNS tissue accelerate disease progression. The underlying mechanisms underlying leukocyte trafficking to the brain has been an active area of research. To recapitulate these observations in pre-clinical mice models, experimental autoimmune encephalitis (EAE) evoked by an injection of myelin proteins is commonly used. Both T and B cells are strongly implicated in MS (Chastain et al., 2011; van Langelaar et al., 2020; Jain and Yong, 2021). Self-reactive T cells in the choroid plexus support neuroimmune homeostasis in the CNS, but in MS and EAE, cerebrospinal fluid T cells skew toward a pathogenic T helper 17 (Th17) phenotype (Mundt et al., 2019) that are detrimental for brain function. Inflamed T leukocytes are more prone to rolling and adhesion along the luminal surface of blood vessels in the brain (Figure 1; Nourshargh and Alon, 2014). “Stickiness” among circulating leukocytes is partly mediated by upregulation of adhesion molecules on the leukocytes themselves, and by alterations in the cell-surface profile of vascular endothelial cells (Pinheiro et al., 2016). Strikingly, upregulation of integrins and cadherins enable Th17 cells to “crawl” for long distances (hundreds of microns) against the direction of blood flow in EAE (Engelhardt and Ransohoff, 2012). During crawling, Th17 cells utilize actin filaments, scaffolding proteins, and ECM-degrading enzymes to infiltrate the brain at fenestrated capillaries in the choroid plexus and penetrate tight junctions at the cerebrovascular interface (Mundt et al., 2019; Samus et al., 2020).

Once in the brain, Th17 cells support neuroinflammation that is the hallmark of MS. T cell diapedesis is evoked by antigen presentation, and in MS/EAE, myelin and myelin-associated proteins are the predominant antigens recognized by T cell receptors (Pinheiro et al., 2016; Mundt et al., 2019). Microglia are the professional antigen-presenting cells (APCs) in the brain parenchyma, but astrocytes are also capable of antigen presentation under certain conditions (Mundt et al., 2019). In the cerebrovasculature, endothelial cells are also capable of antigen presentation in EAE and other chronic inflammatory conditions (Pinheiro et al., 2016). B cells in the dural meninges are also efficient APCs, and they accumulate and skew toward a pro-inflammatory phenotype in EAE (Jain and Yong, 2021). The diversity of cells that can serve as APCs (Chastain et al., 2011) highlight the complicated signaling cascades that underlie neuroinflammation in MS, but also serve as opportunities for therapeutic intervention.

While T cells have historically been the dominant lymphocyte implicated in MS, the role of B cells has received outsized attention in recent years. In a landmark 2017 clinical trial, depletion of CD20+ B cells with the monoclonal antibody Ocrelizumab was identified to be an effective therapy for MS (Hauser et al., 2017). More recently, an epidemiological study of over 10 million active duty U.S. military personnel found strong evidence that Epstein-Barr virus (EBV) serves as the trigger for MS (Bjornevik et al., 2022). In a separate study, it was found that EBV infection triggers B cells to produce antibodies against the EBV transcription factor EBNA1, and strikingly these clones undergo further affinity maturation in the brain to produce auto-antibodies which are also cross-reactive against the CNS protein GlialCAM (Lanz et al., 2022). These consequential studies highlight the potentially detrimental role that mimcry of viral antigens to self-antigens can have in MS and other neuro-inflammatory diseases, and are exciting new avenues to explore for therapeutic interventions.

## 4. Outlook

Traditional concepts on the immuno-separation of the brain from the periphery are slowly being replaced with a more rigorous understanding of the dynamic interactions between the two. The role “borders” such as the BBB play an important role in the dysfunction between neuron-immune cell interactions. As the role of peripheral leukocytes, and in particular T and B cells, in various disease pathologies becomes more evident, new tools will be needed to study and control the circuitry of the immune system in the brain. Cellular and molecular transport across the BBB will play an important role in these therapeutic paradigms. While we have focused on the effect immune cells can have on brain tissue, studying the bi-directional communication between neurons and immune cells will be an important component in neuro-immune therapies. Investigating the immuno-modulatory potential of neurons and bringing together immune tools with bioelectronic medicine is a promising avenue to explore new treatment modalities against hard-to-treat diseases.

## Author Contributions

AT and PA outlined the article. AT, CA, AS, and PA performed literature search and wrote the article. All authors contributed to the article and approved the submitted version.

## Conflict of Interest

The authors declare that the research was conducted in the absence of any commercial or financial relationships that could be construed as a potential conflict of interest.

## Publisher's Note

All claims expressed in this article are solely those of the authors and do not necessarily represent those of their affiliated organizations, or those of the publisher, the editors and the reviewers. Any product that may be evaluated in this article, or claim that may be made by its manufacturer, is not guaranteed or endorsed by the publisher.
